# IKZF3/Aiolos Is Associated with but Not Sufficient for the Expression of IL-10 by CD4^+^ T Cells

**DOI:** 10.4049/jimmunol.1901283

**Published:** 2020-04-22

**Authors:** Michael L. Ridley, Veerle Fleskens, Ceri A. Roberts, Sylvine Lalnunhlimi, Aldana Alnesf, Aoife M. O’Byrne, Kathryn J. A. Steel, Giovanni A. M. Povoleri, Jonathan Sumner, Paul Lavender, Leonie S. Taams

**Affiliations:** * Centre for Inflammation Biology and Cancer Immunology, Department of Inflammation Biology, School of Immunology and Microbial Sciences, King’s College London, London SE1 1UL, United Kingdom;; † Department of Infectious Diseases, School of Immunology and Microbial Sciences, King’s College London, Guy’s Hospital, London SE1 9RT, United Kingdom; and; ‡ MRC and Asthma UK Centre in Allergic Mechanisms of Asthma, Peter Gorer Department of Immunobiology, School of Immunology and Microbial Sciences, King’s College London, Guy’s Hospital, London SE1 9RT, United Kingdom

## Abstract

The expression of anti-inflammatory IL-10 by CD4^+^ T cells is indispensable for immune homeostasis, as it allows T cells to moderate their effector function. We previously showed that TNF-α blockade during T cell stimulation in CD4^+^ T cell/monocyte cocultures resulted in maintenance of IL-10–producing T cells and identified IKZF3 as a putative regulator of IL-10. In this study, we tested the hypothesis that IKZF3 is a transcriptional regulator of IL-10 using a human CD4^+^ T cell–only culture system. IL-10^+^ CD4^+^ T cells expressed the highest levels of IKZF3 both ex vivo and after activation compared with IL-10–CD4^+^ T cells. Pharmacological targeting of IKZF3 with the drug lenalidomide showed that IKZF3 is required for anti-CD3/CD28 mAb–mediated induction of IL-10 but is dispensable for ex vivo IL-10 expression. However, overexpression of IKZF3 was unable to upregulate IL-10 at the mRNA or protein level in CD4^+^ T cells and did not drive the transcription of the *IL10* promoter or putative local enhancer constructs. Collectively, these data indicate that IKZF3 is associated with but not sufficient for IL-10 expression in CD4^+^ T cells.

## Key Points

- Anti-TNF maintains *IL10* expression in CD4^+^ T cells at the transcriptional level.
- IKZF3 is enriched in IL-10^+^ CD4^+^ T cells; degrading IKZF3 disrupts IL-10 production.
- Overexpression of IKZF3 does not drive *IL10* or activate local enhancers.

## Visual Abstract

**Figure:**
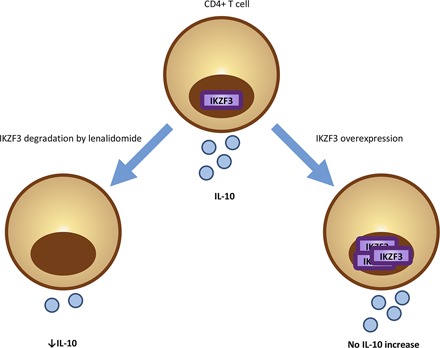

## Introduction

The production of IL-10 by CD4^+^ T cells is key for the control of effector function in response to immune challenge (1–3). Even in the absence of pathogens, CD4^+^ T cell–specific deletions of *Il10* lead to a pronounced inflammation in the colonic mucosa in response to commensal gut bacteria (1).

*IKZF3* (encoding for the protein Aiolos) is a member of the Ikaros Zinc finger family of transcription factors (4). This gene is expressed by various immune cell types and has been implicated in the function of multiple Th subsets (5, 6) as well as in controlling CD4/CD8 fate decision in the thymus (7). The expression of IKZF3 in IL-17–producing CD4^+^ T cells (Th17 cells) is associated with a “nonpathogenic” signature, which includes increased IL-10 production (6, 8). IKZF3 has also been shown to interact with known regulators of *IL10* expression, including its most closely related family member IKZF1 (encoding Ikaros) (4) which has been shown in mice to directly affect the expression of *Il10* (9).

Whereas IKZF3 has been suggested to act as a transcriptional activator in CD4^+^ T cells (4, 10), this has mainly been ascribed to its cooperation with other factors, such as FOXP3 (11) and BLIMP1 in CD4^+^ regulatory T cells (Tregs) (12), and with STAT3 in T follicular helper cells (13). Studies in multiple cell lines highlight the ability of IKZF3 to repress gene expression through HDAC and PRC2 recruitment (14–16) as well as by altering chromatin superstructure (17).

Anti–TNF-α mAb therapy is commonly used in the treatment of many inflammatory conditions, including rheumatoid arthritis (18), inflammatory bowel disease (19), and psoriasis (20). Although the mechanisms governing its therapeutic effects are still not entirely elucidated, multiple effects on the immune system have been reported, including induction of an anti-inflammatory CD4^+^ T cell phenotype (21), modulation of innate immune cell function (22, 23), and expansion of Tregs (24), in addition to blocking TNF-α proinflammatory signaling. We previously demonstrated that patients with rheumatoid arthritis or ankylosing spondylitis treated with anti–TNF-α drugs have increased frequencies of IL-10^+^ CD4^+^ T cells in peripheral blood (10). Furthermore, CD4^+^ T cells from the peripheral blood of healthy volunteers activated in the presence of anti–TNF-α therapeutics had increased frequencies of IL-10^+^ cells (10, 25). Gene expression analysis from one of these studies highlighted IKZF3 as a potential regulator of IL-10 expression, at least in Th17 cells (10).

In this study we sought to address the hypothesis that IKZF3 is a transcriptional regulator of IL-10 production in CD4^+^ T cells.

## Materials and Methods

### Cells and cell culture

Peripheral blood was obtained from healthy adult volunteers with written informed consent (Bromley Research Ethics Committee reference 06/Q0705/20). PBMCs were isolated using density gradient centrifugation. CD4^+^ T cells and CD14^+^ monocytes were isolated by MACS using the manufacturer’s protocol. CD14^+^ monocytes were isolated using anti-CD14^+^ microbeads to ∼98% purity (Miltenyi Biotec), and CD4^+^ T cells were isolated using negative selection to ∼95% purity (Miltenyi Biotec).

Cells were cultured in RPMI 1640 (Life Technologies) supplemented with 10% FCS and 1% penicillin–streptomycin and 10 mg/ml l-glutamine (culture medium). CD4^+^ T cell cultures were stimulated with anti-CD3/CD28 mAb stimulation by coating tissue culture plate wells with 1.25 μg/ml α-CD3 mAb OKT3 (Janssen-Cilag) in PBS for 3 h at 37°C. Wells were washed with sterile PBS before adding the cells (1 × 10^6^ cells/ml) together with 1 μg/ml anti-CD28 mAb (clone CD8.2; BD Biosciences). For cocultures, 0.5 × 10^6^ CD14^+^ peripheral blood monocytes were cultured with 0.5 × 10^6^ autologous CD4^+^ T cells in 1 ml of culture medium in the presence of 100 ng/ml anti-CD3 mAb (OKT3). HEK293T cells (gifted from the Stuart Neil laboratory, King’s College London, London, U.K.) were cultured in DMEM supplemented with 10% FCS, 1% penicillin–streptomycin, and 10 mg/ml l-glutamine. Where indicated, adalimumab (ADA; obtained from Guy’s Hospital Pharmacy) was added at 1 μg/ml.

### Flow cytometry

For intracellular cytokine staining, CD4^+^ T cells or CD4^+^ T cell/monocyte cocultures were stimulated for 3 h in the presence of PMA (50 ng/ml; Sigma-Aldrich), ionomycin (750 ng/ml; Sigma-Aldrich), and GolgiStop (as per the manufacturer’s instructions; BD Biosciences). Cells were washed and stained with CD3-PE Cy7 (UCHT1; BioLegend) and LIVE/DEAD efluor 780 (Thermo Fisher Scientific). Cells were then fixed in 2% PFA and permeabilized with 0.5% saponin (Thermo Fisher Scientific). Cells were subsequently stained for the following cytokines: IL-10–Alexa Fluor 488 (JES3-9D7; BioLegend), IL-17A–PE (BL168; BioLegend), IFN-γ–Pacific blue (4S.B3; BioLegend), and TNF–allophycocyanin (MAb11; BioLegend).

For intranuclear staining of IKZF3, cells were fixed and permeabilized with FOXP3 staining buffer (BioLegend) for 15 min at room temperature before being stained for CD3-PE Cy7, IL-10–Alexa Fluor 488, IL-17A–PE, IFN-γ–Pacific blue, TNF-BV605 (MAb11; BioLegend), and either IKZF3-Alexa Fluor 647 (EPR9342[B]; Abcam) or isotype control (EPR25A; Abcam) for 30 min. Standard gating strategy for intracellular cytokine staining is shown in Supplemental Fig. 1A–C.

### RNA isolation and quantitative PCR

mRNA was isolated using an RNeasy Mini Kit (QIAGEN). cDNA was transcribed using a High-Capacity cDNA Reverse Transcription Kit (Applied Biosystems) according to the manufacturer’s protocol. Real-time PCR was performed using SensiFAST SYBR Green PCR Master Mix (Bioline) with 10 μM primers (Table I). Reactions were performed in multiple technical replicates, and results were calculated using the Δ cycle threshold method.

### Actinomycin D assay

CD4^+^ T cells were stimulated with anti-CD3/CD28 mAb and cultured in the presence or absence of 1 μg/ml ADA for 72 h. After stimulation, the cells were treated for 2 h with either 1 μg/ml actinomycin D (Cambridge Bioscience) or an equivalent volume of DMSO. Cells were subsequently harvested for RNA and assayed for gene expression by quantitative PCR (qPCR).

### Viral transduction of CD4^+^ T cells

The plasmids pCSIG-IKZF3-GFP (lenti-IKZF3) and pCSIG-GFP (lenti-EV) were packaged into lentiviral particles by transfecting HEK293T cells with a pCSIG vector, pSPAX2, and pMD2.G. Viral particles were concentrated using PEG-it (Cambridge Bioscience) according to the manufacturer’s instructions.

Primary CD4^+^ T cells were activated with plate-bound anti-CD3 and anti-CD28 mAb (2 μg/ml) with 20 U/ml recombinant human IL-2 (PeproTech) for 24 h at a density of 1 × 10^6^ cells/ml. Viral supernatants were mixed with TransDux MAX (Cambridge Bioscience), added to the cells, and cultured. After 3 d, the cells were supplemented with fresh 10% FCS RPMI 1640 and 20 U/ml recombinant human IL-2 and rested from stimulation for 3 d. These cells were subsequently sorted on live CD3^+^ GFP^+/−^ cells (Supplemental Fig. 1D). Cells were rested overnight at a density of 1 × 10^5^ cells/ml, then stained for IL-10, IL-17A, IFN-γ, and IKZF3.

### Plasmids and cloning

The selected regions of the human *IL10* locus (indicated in Table II) were amplified by PCR using the BAC RP11-262N9 (Thermo Fisher Scientific) as a template, and TOPO-cloned into pCR-Blunt II-TOPO (Invitrogen). These were then sequenced to confirm 100% conformity to the reference sequence. These regions of interest were subcloned into a pGL4.26 vector (Promega).

FLAG-cMAF-pCMV was a gift from P. Lavender (King’s College London), and HA-IKZF3 was PCR-cloned from a pCMV sport vector purchased from Source BioScience.

### Luciferase assay

HEK293T cells were seeded at a density of 200,000 cells/ml in 96-well plates. On the next day, each well was transfected with 1 μg of polyethylenimine (Sigma-Aldrich) mixed with 0.2 μg of experimental pGL4.26, 0.01 μg of control pRL4, and 0.2 μg of transcription factor-pCSIG or empty vector. After 18 h of transfection, the cell culture media was replaced and left for a further 48 h before harvesting the cells.

Luciferase assays were performed using the Dual-Glo Luciferase Kit (Promega) according to the manufacturer’s instructions, and data were collected on a Tecan Spark 10M. Firefly luciferase activity was normalized to Renilla luciferase activity for each sample to control for transfection efficiency and further normalized to the empty vector control.

### Statistical analysis

Statistical analysis was performed using GraphPad Prism version 8. A Wilcoxon test was used for comparisons between two groups, unless otherwise stated. Significant *p* values are reported as *p < 0.05, **p < 0.01, ***p < 0.001, and ****p < 0.0001.

## Results

### TNF-α blockade maintains IL10 transcription in CD4^+^ T cells

**FIGURE 1.**
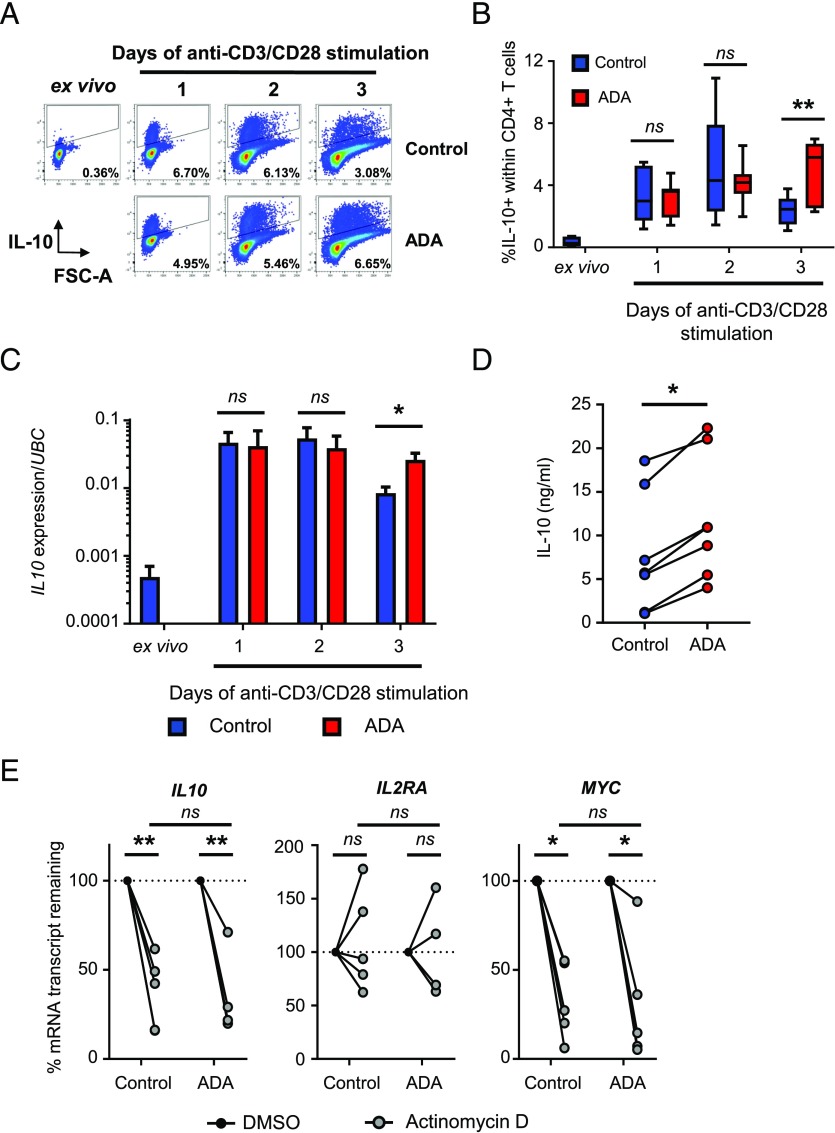
TNF-α blockade maintains the expression of IL-10 in CD4^+^ T cells, which involves active transcription. (**A** and **B**) Primary CD4^+^ T cells from healthy donors were stimulated with anti-CD3/CD28 mAb for 0, 1, 2 or 3 d in the absence (blue bars) or presence (red bars) of 1 μg/ml ADA. Cells were restimulated with PMA and ionomycin and assessed for IL-10 expression. Representative (A) and cumulative (*n* = 7) (B) data showing the frequency of IL-10–expressing cells within CD4^+^ T cells. (**C**) *IL10* mRNA expression was analyzed by qPCR after 1, 2, or 3 d in culture without restimulation (*n* = 6). Data in (B) and (C) were analyzed by two-way ANOVA. (**D**) Quantification of IL-10 in cell culture supernatants from CD4^+^ T cells stimulated as above for 3 d (*n =* 7; Wilcoxon test). (**E**) After 3 d of culture as above, CD4^+^ T cells were treated with either DMSO or 1 μg/ml actinomycin D for 120 min to block transcription. mRNA abundance was assessed by qPCR (*n* = 5; two-way ANOVA with multiple comparisons, comparing DMSO and actinomycin D treatment conditions within each group as well as actinomycin D–treated cells between cells stimulated in the absence or presence of 1 μg/ml ADA). *p < 0.05, **p < 0.01.

We previously observed a transient increase in the frequency of IL-10^+^ CD4^+^ T cells when PBMCs were stimulated with anti-CD3 mAb, which was maintained in the presence of TNF-α blockade (10, 25). Because we aimed to use a reductionist CD4^+^ T cell culture in our experiments, we first sought to determine the kinetics of IL-10 expression in cultures of anti-CD3/CD28 mAb–stimulated CD4^+^ T cells rather than PBMC cultures. CD4^+^ T cells were purified and stimulated with plate-bound anti-CD3 and soluble anti-CD28 mAb for 1–3 d with or without the anti–TNF-α Ab ADA before being restimulated with PMA and ionomycin for intracellular cytokine staining (representative gating strategies are shown in Supplemental Fig. 1). We observed a transient increase in the frequency of IL-10^+^ cells when CD4^+^ T cells were stimulated with anti-CD3/CD28 mAb, which was maintained by TNF-α blockade (Fig. 1A, 1B). To rule out a possible artifact due to the PMA/ionomycin restimulation, we examined the expression of *IL10* mRNA levels by qPCR in CD4^+^ T cells stimulated with anti-CD3/CD28 mAb with or without anti-TNF (Table I). We observed a similar pattern, namely a transient increase of *IL10* expression upon stimulation, which was maintained in the presence of ADA at day 3 (Fig. 1C). We also observed IL-10 secretion in the cell culture supernatant upon 3 d of anti-CD3/CD28 mAb stimulation that was significantly increased in the presence of anti–TNF-α (Fig. 1D).

**Table I.** qPCR primer sequences used in this study

Gene Name	Forward Primer Sequence	Reverse Primer Sequence
*UBC*	5′-CGGGATTTGGGTCGCAGTTCTTG-3′	5′-CGATGGTGTCACTGGGCTCAA-3′
*GAPDH*	5′-GTCAGCCGCATCTTCTTTTGC-3′	5′-AATCCGTTGACTCCGACCTTCC-3′
*B2M*	5′-GTATGCCTGCCGTGTGAAC-3′	5′-AAAGCAAGCAAGCAGAATTTGG-3′
*IL10*	5′-GCCTAACATGCTTCGAGATC-3′	5′-TGATGTCTGGGTCTTGGTTC-3′
*IKZF3*	5′-AGCAGGCCAACCAGTGGAAAGA-3′	5′-TGGGCGTTCACCAGTATGGCT-3′
*IL2RA*	5′-ACAAGCTCTGCCACTCGGAAC-3′	5′-AGCCCTGTATCCCTGGACG-3′
*MYC*	5′-TAGTGGAAAACCAGCCTCCC-3′	5′-GGCAGCAGCTCGAATTTCTT-3′

**Table II.** UCSC Genome Browser hg19 coordinates for regions used in cloning putative *IL10* enhancer and promoter regions

Region Name	Region Coordinates (hg19) Chr1	Forward Primer Sequence	Reverse Primer Sequence
1	206907901–206908425	5′-TTGCTAGCTTTTGCCAAATGCAGAATCA-3′	5′-AACTCGAGAAGGGGACAAGCAGGTCTCT-3′
2	206910301–206910900	5′-AACTCGAGAAGGGGACAAGCAGGTCTCT-3′	5′-TTGCTAGCAAAAGTGCTGGGCTTACAGG-3′
3	206912926–206913525	5′-TTGCTAGCAAAAGTGCTGGGCTTACAGG-3′	5′-AACTCGAGGGGAGCCACACTGTTCTGAC-3′
4	206930851–206931750	5′-AACTCGAGGGGAGCCACACTGTTCTGAC-3′	5′-TTGCTAGCTTTCCAGGACCCTGATATGC-3′
5	206939626–206940225	5′-TTGCTAGCTTTCCAGGACCCTGATATGC-3′	5′-AACTCGAGTGCTTTCATCCATCCAACAG-3′
6	206942701–206943150	5′-AACTCGAGTGCTTTCATCCATCCAACAG-3′	5′-TTGCTAGCTCACTGGTCACAGGACATCAC-3′
Promoter	206945773–206947172	5′-TTGCTAGCTCACTGGTCACAGGACATCAC-3′	5′-AACTCGAGATTTAGTCGGGTGTGGTGGT-3′
7	206957626–206958150	5′-AACTCGAGATTTAGTCGGGTGTGGTGGT-3′	5′-TTGCTAGCCCTGAGTAGTTCCCAGCTTGA-3′
8	206964826–206965275	5′-TTGCTAGCCCTGAGTAGTTCCCAGCTTGA-3′	5′-AACTCGAGTGAGCTAACAGGGCTTCTGG-3′
9	206976226–206977275	5′-AACTCGAGTGAGCTAACAGGGCTTCTGG-3′	5′-TTGCTAGCAGGTGCGAAGGGCAGAT-3′
10	206990176–206990550	5′-TTGCTAGCAGGTGCGAAGGGCAGAT-3′	5′-AACTCGAGAGCAGGTGAAGAATGCCTTT-3′

*IL10* mRNA has been shown previously to be controlled at the posttranscriptional level (26). To determine whether *IL10* mRNA was stabilized by TNF-α blockade, we performed an actinomycin D assay on CD4^+^ T cells stimulated with anti-CD3/CD28 mAb for 3 d. This assay is frequently used to determine the relative stability of mRNA species between treatments or cell types (27). The treatment of cells with actinomycin D inhibits mRNA transcription. Once blocked, unstable mRNA transcripts are degraded by cellular machinery over time and not replenished. Comparing mRNA levels between actinomycin D and vehicle control-treated cells gives an indication of mRNA stability. *IL10* mRNA in activated CD4^+^ T cells was sensitive to the addition of actinomycin D and therefore unstable, similar to *MYC* and unlike the more stable mRNA *IL2RA* (Fig. 1E). We did not observe a significant difference between control- and ADA-treated CD4^+^ T cells. These results indicate that the increase in *IL10* mRNA is due to active transcription.

### IKZF3 is enriched in IL-10–producing CD4^+^ T cells

**FIGURE 2.**
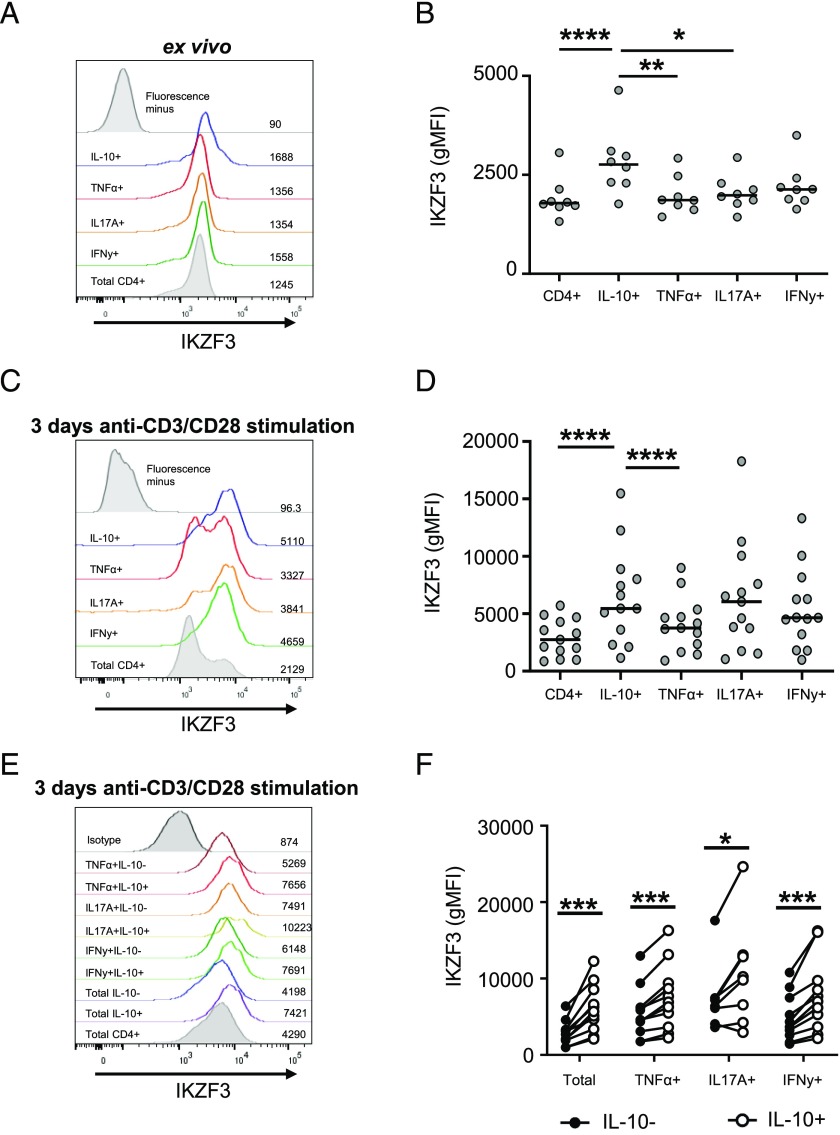
IKZF3 is associated with IL-10–producing CD4^+^ T cells. (**A** and **B**) Primary CD4^+^ T cells from healthy donors were stimulated with PMA and ionomycin and assessed for frequency of cytokine-producing cells and IKZF3 expression. Representative gMFI of IKZF3 expression shown on the right of each histogram (A) and cumulative data (*n* = 8) shown (B). (**C**–**F**) CD4^+^ T cells were stimulated with anti-CD3/CD28 mAb for 3 d and subsequently restimulated with PMA and ionomycin and assessed for frequency of cytokine-producing cells and IKZF3 expression. Expression of IKZF3 was calculated within total populations of cytokine-producing cells (C and D) or within IL-10^+^ or IL-10^−^ subsets within those populations (E and F). Representative gMFI of IKZF3 expression shown on the right of each histogram (C) and cumulative (*n*=13) (D) data of total cytokine-producing populations are shown. Representative gMFI of IKZF3 expression shown on the right of each histogram (E) and cumulative (*n* = 8–11) (F) data for IKZF3 expression within IL-10^+^ and IL-10^−^ subsets are shown. Data in (B) and (D) were analyzed by ANOVA, data in (F) was analyzed by Wilcoxon test. *p < 0.05, **p < 0.01, ***p < 0.001, ****p < 0.0001.

Our previous gene expression analysis indicated that IKZF3 was upregulated in Th17 cells in response to TNF-α blockade and could bind at the *IL10* locus in these cells (10). To examine whether IKZF3 was associated with IL-10 production in CD4^+^ T cells, we performed a combined intracellular cytokine staining and an intranuclear stain for IKZF3 to determine the expression of IKZF3 within CD4^+^ T cells expressing IL-10, IL-17A, IFN-γ, or TNF-α, either ex vivo or after 3 d of anti-CD3/CD28 mAb stimulation. IKZF3 was expressed at higher levels in IL-10^+^ CD4^+^ T cells compared with the total CD4^+^ T cell population and the IL-17A^+^ and TNF-α^+^ subsets ex vivo (Fig. 2A, 2B). Upon anti-CD3/CD28 mAb stimulation, a significant increase was observed in IKZF3 expression in IL-10−expressing cells compared with the total CD4^+^ and the TNF-α^+^ cell populations. However, there was no longer a significant difference between IL-10^+^ and IL-17A^+^ CD4^+^ T cells (Fig. 2C, 2D). Because IL-10 can be expressed by multiple cytokine-producing CD4^+^ T cell subsets (especially after stimulation), we compared IKZF3 expression in the IL-17A^+^, TNF-α^+^, and IFN-γ^+^ cells that coproduced IL-10 and those that did not (Fig. 2E, 2F). In all subsets analyzed, a significantly higher expression of IKZF3 was observed in IL-10–coproducing CD4^+^ T cells compared with cells that did not produce IL-10 (Fig. 2F).

In our previous work, we observed an increase in IKZF3 expression in Th17 cells following TNF-α blockade using a CD14^+^ monocyte/CD4^+^ T cell coculture system. To determine whether the increase in IKZF3 upon TNF-α blockade occurred in the absence of monocytes and in all T cell subsets, we compared IKZF3 expression in CD4^+^ T cells cultured alone versus CD4^+^ T cells cocultured with CD14^+^ monocytes in the absence or presence of ADA (Supplemental Fig. 2). We previously established that IL-10 expression is increased upon TNF blockade in both culture systems (10, 25). In agreement with our previous results, upon T cell stimulation in the presence of CD14^+^ monocytes and anti-TNF, IKZF3 expression was increased in the total CD4^+^ T cell population as well as in the IL-10^+^ and IL-17A^+^ subsets (Supplemental Fig. 2A). In the absence of CD14^+^ monocytes, IL-10^+^ CD4^+^ T cells had high expression of IKZF3 in both control- and ADA-treated samples, but TNF-α blockade did not alter IKZF3 expression in these cells (Supplemental Fig. 2B, 2C). These data indicate that in CD4^+^ T cell–only cultures, the anti–TNF-α–mediated increase of IL-10 can occur in the absence of a concomitant increase in IKZF3 expression.

### IKZF3 degradation by lenalidomide does not alter IL-10 expression ex vivo but disrupts anti-CD3/CD28 mAb–mediated IL-10 production

We sought to determine whether IKZF3 is required for IL-10 expression. We first attempted to deplete IKZF3 from CD4^+^ T cells using small interfering RNA in primary CD4^+^ T cells. However, this approach did not work because of the stability of the IKZF3 protein (as shown by cycloheximide assays; data not shown) and its upregulation upon anti-CD3/CD28 stimulation (required to render the cells transfectable or transducable; data not shown). As an alternative approach, we employed the thalidomide derivative lenalidomide, which has been shown to induce the proteasomal degradation of IKZF3 (and IKZF1) and is used therapeutically in treating multiple myeloma (28–30).

**FIGURE 3.**
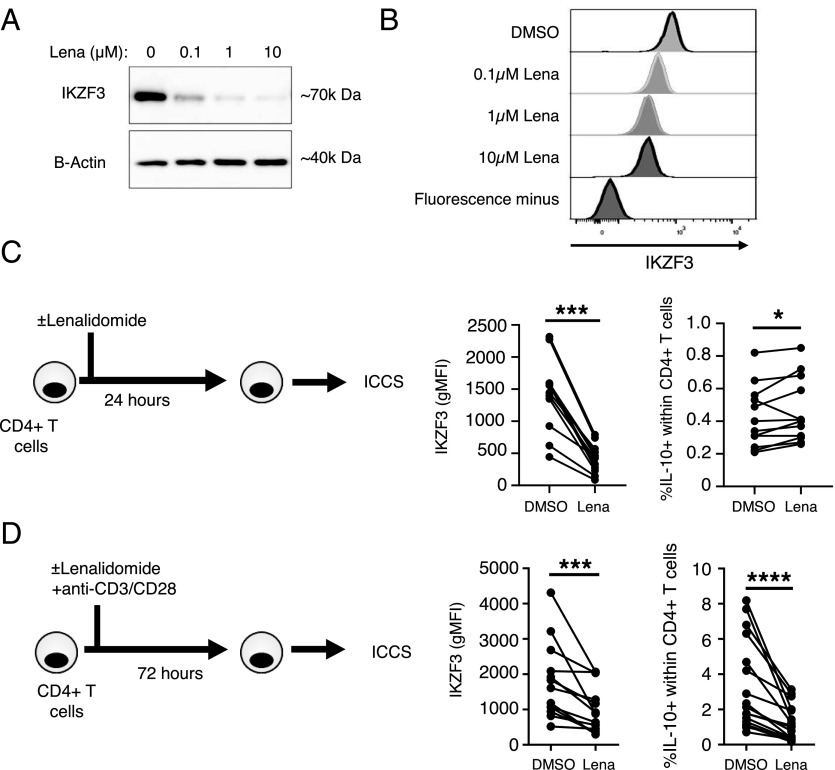
Lenalidomide disrupts the anti-CD3/CD28 mAb–mediated induction of IL-10^+^ CD4^+^ T cells but does not affect ex vivo IL-10 production. (**A** and **B**) Primary CD4^+^ T cells from healthy donors were treated with 0.1, 1, or 10 μM lenalidomide overnight and examined for IKZF3 expression by Western blot (A) or flow cytometry (B). (**C**) CD4^+^ T cells were treated with 1 μM lenalidomide overnight and then stimulated with PMA and ionomycin and assessed for IKZF3 expression and frequency of IL-10^+^ CD4^+^ T cells (*n* = 13). (**D**) CD4^+^ T cells were stimulated with anti-CD3/CD28 mAb for 3 d in the presence of 1 μM lenalidomide and subsequently restimulated with PMA and ionomycin and assessed for IKZF3 expression and frequency of IL-10^+^ CD4^+^ T cells (*n* = 13). Data in (C) and (D) were analyzed by Wilcoxon test. *p < 0.05, ***p < 0.001, ****p < 0.0001. ICCS, intracellular cytokine staining.

Treatment of CD4^+^ T cells with lenalidomide overnight led to a dose-dependent decrease in IKZF3 protein levels as shown by Western blot (Fig. 3A) and flow cytometry (Fig. 3B). CD4^+^ T cells were then treated with lenalidomide for 24 h in the absence of T cell activation followed by intracellular cytokine staining. Although a significant reduction in the levels of IKZF3 was observed, the frequency of IL-10^+^ cells within CD4^+^ T cells was slightly increased (Fig. 3C). Ex vivo treatment of CD4^+^ T cells with lenalidomide had no effect on IL-17A and IFN-γ expression or viability (Supplemental Fig. 3A). In contrast, when CD4^+^ T cells were treated with lenalidomide for 3 d in the presence of anti-CD3/CD28 mAb stimulation, a strong reduction in both IKZF3 expression and the frequency of IL-10^+^ CD4^+^ T cells was observed (Fig. 3D). These data indicate that, whereas IL-10 production in unstimulated CD4^+^ T cells is not lenalidomide-sensitive, the anti-CD3/CD28 mA–mediated increase in IL-10–expressing cells is lenalidomide-sensitive and thus, by extrapolation, potentially regulated by IKZF3. Lenalidomide treatment for 72 h also resulted in statistically significant increases in IFN-γ^+^ and TNF-α^+^ frequencies, a decrease in IL-17A^+^ frequencies, and a slight decrease in cell viability (median viability: 88.9–82.75%, control versus lenalidomide, respectively; Supplemental Fig. 3B). Treatment of CD4^+^ T cells with lenalidomide also consistently increased secretion of IL-2 by CD4^+^ T cells after 3 d of anti-CD3/CD28 mAb stimulation (Supplemental Fig. 3C, *n* = 5).

### IKZF3 is not sufficient to drive expression of IL10 in CD4^+^ T cells at the mRNA or protein level

**FIGURE 4.**
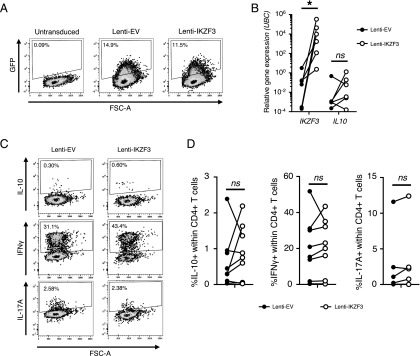
IKZF3 overexpression in CD4^+^ T cells is not sufficient to drive the expression of IL-10. (**A**–**D**) Primary CD4^+^ T cells from healthy donors were transduced with an IKZF3-IRES-GFP (lenti-IKZF3) or GFP-only (lenti-EV) lentivirus. Seven days posttransduction, cells were sorted on GFP expression. (A) Representative GFP expression. (B) CD4^+^ T cells transduced with lenti-IKZF3 or lenti-EV were sorted on GFP expression, and mRNA expression of *IKZF3* and *IL10* was quantified by qPCR (*n* = 6). (C and D) Cells transduced with lenti-IKZF3 or lenti-EV were sorted on GFP expression, rested overnight, then restimulated with PMA and ionomycin and assessed for frequency of IL-10, IFN-γ, or IL-17A–producing cells. Representative (C) and cumulative (*n* = 6–8) (D) data are shown. Data in (B) and (D) were analyzed by Wilcoxon test. *p < 0.05.

We next sought to determine whether IKZF3 was sufficient to drive IL-10 expression in CD4^+^ T cells. To overexpress IKZF3, we activated CD4^+^ T cells and transduced the cells with an IKZF3-IRES-GFP lentiviral construct (lenti-IKZF3) or an empty vector (lenti-EV) encoding only GFP (Fig. 4A). After transduction, live GFP^+^ cells were sorted for mRNA isolation or rested and stimulated with PMA/ionomycin for intracellular cytokine staining. Although cells transduced with IKZF3 showed a significant increase in *IKZF3* transcript, *IL10* mRNA levels were low and not consistently increased by IKZF3 overexpression (Fig. 4B). Also, at the protein level, IKZF3-transduced cells did not show a consistent increase in IL-10–producing cells compared with the empty vector (Fig. 4C, 4D). A considerable proportion of cells was able to produce IFN-γ or IL-17A, indicating that the transduction protocol had not affected the capacity of the cells to produce cytokines. Together, these data indicate that IKZF3 overexpression is not sufficient to drive IL-10 expression in CD4^+^ T cells.

### IKZF3 is insufficient to drive the expression of enhancer or promoter elements of IL-10

**FIGURE 5.**
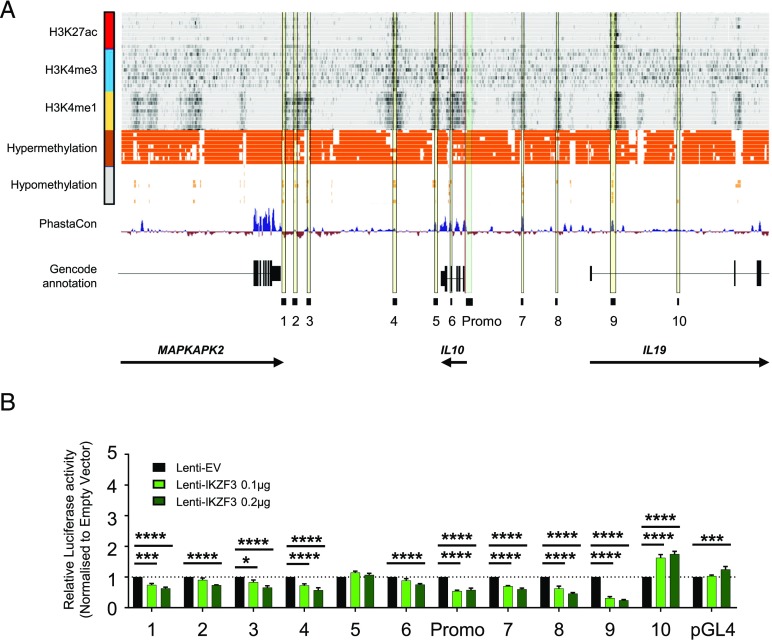
IKZF3 is insufficient to drive transcriptional activity via the *IL10* promoter or local enhancers. (**A**) Chromatin immunoprecipitation sequencing (from the BLUEPRINT consortium) and assay for transposase-accessible chromatin using sequencing (ATAC-seq) data (from Ref. 31) from ex vivo human CD4^+^ T cells for the *MAPKAPK2:IL10:IL19* locus was used to identify regions of putative enhancers (yellow vertical bars numbered 1–10) as well as 1.50-kb promoter region of *IL10* (vertical green bar labeled Promo). (**B**) These promoter and enhancer regions were then cloned upstream of a luciferase reading frame and transfected along with an expression plasmid encoding IKZF3 (lenti-IKZF3) or empty vector control (lenti-EV) into HEK293T cells and assessed 48 h later for luciferase activity (*n* = 4). Data were analyzed by two-way ANOVA. *p < 0.05, ***p < 0.001, ****p < 0.0001.

Our previous work showed that IKZF3 is able to bind evolutionarily conserved regions at the *IL10* locus in Th17 cells. To determine whether IKZF3 can drive transcription of *IL10* via these regions, we identified 10 putative enhancer sites at the *IL10* locus (Fig. 5A), as defined by accessible chromatin (31), high H3K4me1, and low CpG methylation [from the BLUEPRINT consortium (32)]. We cloned these regions (genomic coordinates of cloned regions defined in Table II) and a 1.5-kb region of the *IL10* promoter upstream of a Firefly luciferase open reading frame (pGL4). These vectors were then cotransfected with a control Renilla luciferase vector together with the plasmids lenti-IKZF3 (Fig. 5B) or lenti-MAF (Supplemental Fig. 4B, 4C), a known regulator of *IL10* (33). To validate that our constructs were functional, we stained HEK293T cells transfected with lenti-IKZF3, lenti-MAF, or lenti-EV for IKZF3 or cMAF by flow cytometry (Supplemental Fig. 4A, 4B) and observed at least a 10-fold increase in expression in the relevant conditions.

The luciferase experiments showed that IKZF3 has limited capacity to drive transcription of the *IL10* constructs (Fig. 5B). An induction of reporter gene expression in response to IKZF3 transfection was only seen for enhancer 10, whereas reporter gene expression for most other constructs decreased in a dose-dependent manner upon increasing amounts of IKZF3. In contrast, transfection with cMAF, a known transcriptional regulator of *IL10* (34, 35), significantly upregulated multiple enhancers compared with the empty vector (Supplemental Fig. 4C).

## Discussion

Regulation of IL-10 expression is a multilayered process at the levels of transcription (33, 36), posttranscriptional stability (37, 38), and translation (39). In the innate immune system, IL-10 has been shown to be temporally regulated through regulation of transcript stability, such as through the p38/TTP axis (26, 37).

We found that *IL10* mRNA was maintained at higher levels in the presence of anti–TNF-α mAb. This increase in IL-10–producing CD4^+^ T cells does not appear to be attributable to changes in cell survival or increased cell proliferation after TNF blockade, as we showed recently (40).

We also show that *IL10* mRNA in anti-CD3/CD28 mAb–activated primary human CD4^+^ T cells is an unstable transcript. This may represent a mechanism by which CD4^+^ T cells, which can transiently produce IL-10 on stimulation, eventually prevent its expression via negative feedback, similar to macrophages (41).

To understand what drives the transcriptional regulation of *IL10*, we focused on IKZF3. Our previous work with a CD4^+^ T cell:CD14^+^ monocyte coculture system showed increased IKZF3 expression upon TNF-α blockade in Th17 cells, which correlated with increased IL-10 expression. In our current study using a T cell reductionist system, we saw no change in IKZF3 expression in cytokine-producing CD4^+^ T cell subsets upon TNF-α blockade, although we did observe an increase in IL-10^+^ CD4^+^ T cell frequency. We did observe a generally higher level of IKZF3 expression in IL-10–producing CD4^+^ T cells ex vivo and after CD3/CD28 stimulation. An association between IKZF3 and IL-10–producing CD4^+^ T cells has been noted by other studies in human Th17 clones (42) as well as mouse Th1 (5) and Th17 cells (8). This association may indicate common transcriptional regulators under steady-state conditions but not upon TNF-α blockade. In our study, IKZF3 was highly expressed in IL-17A^+^ IL-10^+^ CD4^+^ T cells. The expression of IKZF3 and IL-10 in nonpathogenic Th17 cells with a reduced capacity to drive experimental autoimmune encephalomyelitis has been previously noted (6, 8).

Similar to our findings with CD4^+^ T cells cultured without monocytes, another study found that memory CD4^+^ T cells activated by anti-CD3/CD28 mAb in the presence of the TNF-α inhibitor drug etanercept, in the absence of monocytes, showed an increased expression of *IL10* upon TNF-α blockade that was not accompanied by changes in IKZF3 expression (43).

IKZF3 (and IKZF1) has been previously described as a negative regulator of *Il2* expression in CD4^+^ T cells (6, 29), and our findings that IL-2 secretion is increased upon lenalidomide treatment support that observation. The expression of IL-10 and a reduced capacity to produce IL-2 is a known hallmark of Tregs. Therefore, high IKZF3 expression in the IL-10^+^ population might be indicative of a high proportion of Tregs. However, mass cytometry data from our laboratory did not reveal a higher expression of IKZF3 in CD25^high^ CD127^low^ Tregs compared with CD25^low^ CD127^high^ effector T cells ex vivo (data not shown). Furthermore, upon TNF-α blockade we did not observe an increase in FOXP3^+^ Tregs (10).

Studies have implicated IL-2 signaling to be required for IL-10 expression by multiple Th subsets in vitro (L. Gabryšová, E.H. Mann, L. Bradley, J.I. MacRae, C. Whicher, C.M. Hawrylowicz, D. Anastasiou, and A. O’Garra, manuscript posted on bioRxiv). Therefore, one could expect that blocking IKZF3, which is an *IL2* transcriptional repressor, would lead to an increase in IL-10 production and frequency. However, we instead observed a significant reduction in the frequency of IL-10^+^ CD4^+^ T cells in the presence of lenalidomide.

From our data, lenalidomide also seems to have effects on the frequency of IL-17A-, TNF-α–, and IFN-γ–producing cells. The reduction in IL-17A^+^ CD4^+^ T cells could be due to the increase of IL-2 in the cell culture supernatants, which has been shown to inhibit the differentiation of Th17 cells (6, 44, 45). IL-2 has also been shown to increase the expression of IFN-γ in human CD4^+^ T cells (46, 47) and TNF-α expression in mouse CD8^+^ T cells (48). It should be noted, however, that expression of IFN-γ and TNF-α can be suppressed by IL-10 (49). Therefore, the decrease in IL-10 expression accompanying lenalidomide treatment could boost the induction of IFN-γ–and TNF-α–producing cells.

It should be considered that the effect of lenalidomide on IL-10 production in CD4^+^ T cells may be due to off target effects. Lenalidomide has been shown to downregulate several proteins, including transcription factors (50–52). Therefore, the reduction in anti-CD3/CD28 mAb–induced IL-10 production may stem from another lenalidomide-sensitive protein rather than IKZF3. IKZF1 has previously been shown to be affected by lenalidomide and is capable of binding similar motifs to IKZF3. However, we previously observed no effect of anti-TNF on CD4^+^ T cell expression of IKZF1 (10) and do not see the same association of IKZF1 with IL-10 ex vivo.

To determine whether IKZF3 expression was sufficient to drive IL-10 expression, we overexpressed this protein in primary CD4^+^ T cells to determine its ability to drive *IL10* mRNA and protein expression, as well as in the HEK293T cell line to determine whether it could drive expression of putative *IL10* enhancers or promoters. In both experimental approaches, we found that IKZF3 overexpression was not sufficient to drive the expression of IL-10. IKZF3 may require cofactors to promote transcription, such as BLIMP1 (12) and STAT3 (13), which have been shown to interact with IKZF3. Encyclopedia of DNA Elements (ENCODE) data show that DNA binding motifs of these factors are in similar locations to IKZF family consensus motifs at the *IL10* locus. It may be that these cofactors are not available in transduced CD4^+^ T cells or in HEK293T cells to facilitate *IL10* mRNA or reporter expression.

The effect of IKZF3 on most of the luciferase reporters is consistent with its reported function as a transcriptional repressor and indicates that IKZF3 is unable to directly drive *IL10* expression, even when enhancers and promoters are accessible to bind (6, 15). These reporters were based on assay for transposase-accessible chromatin using sequencing (ATAC-seq) data (31), which should be reflective of the accessible regions in CD4^+^ T cells ex vivo. Changes to chromatin by anti-CD3/CD28 mAb stimulation, however, could reveal other enhancers that IKZF3 can bind to drive expression.

It should be noted that IKZF3 has a number of splice variants that have various abilities to drive gene expression (15). Our data suggest that CD4^+^ T cells predominantly express the largest isoform of ∼70 kDa, and this is the isoform we cloned in our overexpression studies. This isoform has previously been shown to drive gene expression in mouse T follicular helper cell–like cells, and it is therefore possible that this isoform could drive transcription in human CD4^+^ T cells (13). However, we cannot rule out that other IKZF3 isoforms may differentially affect *IL10* expression.

In summary, this study shows that IKZF3 expression is associated with IL-10^+^ CD4^+^ T cells at the protein level and that pharmacological inhibition of IKZF3 disrupts the ability of CD4^+^ T cells to produce IL-10. However, the expression of IKZF3 is not sufficient to drive IL-10 protein or mRNA expression. We also note that, whereas TNF-α blockade does lead to increased *IL10* mRNA expression, this is not necessarily attributable to differential expression of IKZF3. Further work is required to establish the transcription factors modified by TNF-α blockade that lead to increased *IL10* expression and whether such transcriptional regulation occurs in patients treated with TNF-α inhibitors.

## Supplementary Material

Data Supplement

## References

[1] Roers A. Siewe L. Strittmatter E. Deckert M. Schlüter D. Stenzel W. Gruber A. D. Krieg T. Rajewsky K. Müller W. 2004 T cell-specific inactivation of the interleukin 10 gene in mice results in enhanced T cell responses but normal innate responses to lipopolysaccharide or skin irritation. J. Exp. Med. 200: 1289–1297. 15534372 10.1084/jem.20041789 PMC2211912

[2] Jankovic D. Kugler D. G. Sher A. 2010 IL-10 production by CD4+ effector T cells: a mechanism for self-regulation. Mucosal Immunol. 3: 239–246. 20200511 10.1038/mi.2010.8 PMC4105209

[3] Charbonnier L.-M. Han W. G. H. Quentin J. Huizinga T. W. J. Zwerina J. Toes R. E. M. Jorgensen C. Louis-Plence P. 2010 Adoptive transfer of IL-10-secreting CD4+CD49b+ regulatory T cells suppresses ongoing arthritis. J. Autoimmun. 34: 390–399. 19880290 10.1016/j.jaut.2009.10.003

[4] Morgan B. Sun L. Avitahl N. Andrikopoulos K. Ikeda T. Gonzales E. Wu P. Neben S. Georgopoulos K. 1997 Aiolos, a lymphoid restricted transcription factor that interacts with Ikaros to regulate lymphocyte differentiation. EMBO J. 16: 2004–2013. 9155026 10.1093/emboj/16.8.2004 PMC1169803

[5] Yu F. Sharma S. Jankovic D. Gurram R. K. Su P. Hu G. Li R. Rieder S. Zhao K. Sun B. Zhu J. 2018 The transcription factor Bhlhe40 is a switch of inflammatory versus antiinflammatory Th1 cell fate determination. J. Exp. Med. 215: 1813–1821. 29773643 10.1084/jem.20170155 PMC6028509

[6] Quintana F. J. Jin H. Burns E. J. Nadeau M. Yeste A. Kumar D. Rangachari M. Zhu C. Xiao S. Seavitt J. 2012 Aiolos promotes TH17 differentiation by directly silencing Il2 expression. [Published erratum appears in 2014 *Nat. Immunol.* 15: 109.] Nat. Immunol. 13: 770–777. 22751139 10.1038/ni.2363 PMC3541018

[7] Mitchell J. L. Seng A. Yankee T. M. 2016 Expression patterns of Ikaros family members during positive selection and lineage commitment of human thymocytes. Immunology 149: 400–412. 27502439 10.1111/imm.12657 PMC5095499

[8] Lee Y. Awasthi A. Yosef N. Quintana F. J. Xiao S. Peters A. Wu C. Kleinewietfeld M. Kunder S. Hafler D. A. 2012 Induction and molecular signature of pathogenic TH17 cells. Nat. Immunol. 13: 991–999. 22961052 10.1038/ni.2416 PMC3459594

[9] Umetsu S. E. Winandy S. 2009 Ikaros is a regulator of Il10 expression in CD4+ T cells. J. Immunol. 183: 5518–5525. 19828627 10.4049/jimmunol.0901284 PMC2778601

[10] Evans H. G. Roostalu U. Walter G. J. Gullick N. J. Frederiksen K. S. Roberts C. A. Sumner J. Baeten D. L. Gerwien J. G. Cope A. P. 2014 TNF-α blockade induces IL-10 expression in human CD4+ T cells. Nat. Commun. 5: 3199. 24492460 10.1038/ncomms4199 PMC3918582

[11] Kwon H.-K. Chen H.-M. Mathis D. Benoist C. 2017 Different molecular complexes that mediate transcriptional induction and repression by FoxP3. Nat. Immunol. 18: 1238–1248. 28892470 10.1038/ni.3835 PMC5679728

[12] Wei X. Zhang J. Gu Q. Huang M. Zhang W. Guo J. Zhou X. 2017 Reciprocal expression of IL-35 and IL-10 defines two distinct effector Treg subsets that are required for maintenance of immune tolerance. Cell Rep. 21: 1853–1869. 29141218 10.1016/j.celrep.2017.10.090

[13] Read K. A. Powell M. D. Baker C. E. Sreekumar B. K. Ringel-Scaia V. M. Bachus H. Martin R. E. Cooley I. D. Allen I. C. Ballesteros-Tato A. Oestreich K. J. 2017 Integrated STAT3 and Ikaros Zinc finger transcription factor activities regulate Bcl-6 expression in CD4^+^ Th cells. J. Immunol. 199: 2377–2387. 28848064 10.4049/jimmunol.1700106 PMC5657606

[14] Koipally J. Renold A. Kim J. Georgopoulos K. 1999 Repression by Ikaros and Aiolos is mediated through histone deacetylase complexes. EMBO J. 18: 3090–3100. 10357820 10.1093/emboj/18.11.3090 PMC1171390

[15] Caballero R. Setien F. Lopez-Serra L. Boix-Chornet M. Fraga M. F. Ropero S. Megias D. Alaminos M. Sanchez-Tapia E. M. Montoya M. C. 2007 Combinatorial effects of splice variants modulate function of Aiolos. J. Cell Sci. 120: 2619–2630. 17646674 10.1242/jcs.007344

[16] Liang Z. Brown K. E. Carroll T. Taylor B. Vidal I. F. Hendrich B. Rueda D. Fisher A. G. Merkenschlager M. 2017 A high-resolution map of transcriptional repression. eLife 6: e22727. 10.7554/eLife.22767 PMC5373822 28318487

[17] Li X. Xu Z. Du W. Zhang Z. Wei Y. Wang H. Zhu Z. Qin L. Wang L. Niu Q. 2014 Aiolos promotes anchorage independence by silencing p66Shc transcription in cancer cells. Cancer Cell 25: 575–589. 24823637 10.1016/j.ccr.2014.03.020 PMC4070880

[18] Taylor P. C. Feldmann M. 2009 Anti-TNF biologic agents: still the therapy of choice for rheumatoid arthritis. Nat. Rev. Rheumatol. 5: 578–582. 19798034 10.1038/nrrheum.2009.181

[19] Cohen B. L. Sachar D. B. 2017 Update on anti-tumor necrosis factor agents and other new drugs for inflammatory bowel disease. BMJ 357: j2505. 28630047 10.1136/bmj.j2505

[20] Yost J. Gudjonsson J. E. 2009 The role of TNF inhibitors in psoriasis therapy: new implications for associated comorbidities. F1000 Med. Rep. 1: 30. 10.3410/M1-30 PMC2924720 20948750

[21] Boks M. A. Kager-Groenland J. R. Mousset C. M. van Ham S. M. ten Brinke A. 2014 Inhibition of TNF receptor signaling by anti-TNFα biologicals primes naïve CD4(+) T cells towards IL-10(+) T cells with a regulatory phenotype and function. Clin. Immunol. 151: 136–145. 24568737 10.1016/j.clim.2014.02.008

[22] Pallai A. Kiss B. Vereb G. Armaka M. Kollias G. Szekanecz Z. Szondy Z. 2016 Transmembrane TNF-α reverse signaling inhibits lipopolysaccharide-induced proinflammatory cytokine formation in macrophages by inducing TGF-β: therapeutic implications. J. Immunol. 196: 1146–1157. 26729808 10.4049/jimmunol.1501573

[23] Bloemendaal F. M. Koelink P. J. van Schie K. A. Rispens T. Peters C. P. Buskens C. J. van der Bilt J. D. Bemelman W. A. Korf H. Sabino J. G. 2018 TNF-anti-TNF immune complexes inhibit IL-12/IL-23 secretion by inflammatory macrophages via an Fc-dependent mechanism. J. Crohn’s Colitis 12: 1122–1130. 29860435 10.1093/ecco-jcc/jjy075

[24] Nguyen D. X. Ehrenstein M. R. 2016 Anti-TNF drives regulatory T cell expansion by paradoxically promoting membrane TNF-TNF-RII binding in rheumatoid arthritis. J. Exp. Med. 213: 1241–1253. 27270893 10.1084/jem.20151255 PMC4925013

[25] Roberts C. A. Durham L. E. Fleskens V. Evans H. G. Taams L. S. 2017 TNF blockade maintains an IL-10^+^ phenotype in human effector CD4^+^ and CD8^+^ T cells. Front. Immunol. 8: 157. 28261215 10.3389/fimmu.2017.00157 PMC5309392

[26] Stoecklin G. Tenenbaum S. A. Mayo T. Chittur S. V. George A. D. Baroni T. E. Blackshear P. J. Anderson P. 2008 Genome-wide analysis identifies interleukin-10 mRNA as target of tristetraprolin. J. Biol. Chem. 283: 11689–11699. 18256032 10.1074/jbc.M709657200 PMC2431067

[27] Harrold S. Genovese C. Kobrin B. Morrison S. L. Milcarek C. 1991 A comparison of apparent mRNA half-life using kinetic labeling techniques vs decay following administration of transcriptional inhibitors. Anal. Biochem. 198: 19–29. 1789423 10.1016/0003-2697(91)90500-s

[28] Krönke J. Udeshi N. D. Narla A. Grauman P. Hurst S. N. McConkey M. Svinkina T. Heckl D. Comer E. Li X. 2014 Lenalidomide causes selective degradation of IKZF1 and IKZF3 in multiple myeloma cells. Science 343: 301–305. 24292625 10.1126/science.1244851 PMC4077049

[29] Gandhi A. K. Kang J. Havens C. G. Conklin T. Ning Y. Wu L. Ito T. Ando H. Waldman M. F. Thakurta A. 2014 Immunomodulatory agents lenalidomide and pomalidomide co-stimulate T cells by inducing degradation of T cell repressors Ikaros and Aiolos via modulation of the E3 ubiquitin ligase complex CRL4(CRBN.). Br. J. Haematol. 164: 811–821. 24328678 10.1111/bjh.12708 PMC4232904

[30] Lu G. Middleton R. E. Sun H. Naniong M. Ott C. J. Mitsiades C. S. Wong K.-K. Bradner J. E. Kaelin W. G. Jr 2014 The myeloma drug lenalidomide promotes the cereblon-dependent destruction of Ikaros proteins. Science 343: 305–309. 24292623 10.1126/science.1244917 PMC4070318

[31] Buenrostro J. D. Giresi P. G. Zaba L. C. Chang H. Y. Greenleaf W. J. 2013 Transposition of native chromatin for fast and sensitive epigenomic profiling of open chromatin, DNA-binding proteins and nucleosome position. Nat. Methods 10: 1213–1218. 24097267 10.1038/nmeth.2688 PMC3959825

[32] Adams D. Altucci L. Antonarakis S. E. Ballesteros J. Beck S. Bird A. Bock C. Boehm B. Campo E. Caricasole A. 2012 BLUEPRINT to decode the epigenetic signature written in blood. Nat. Biotechnol. 30: 224–226. 22398613 10.1038/nbt.2153

[33] Gabryšová L. Howes A. Saraiva M. O’Garra A. 2014 The regulation of IL-10 expression. In Interleukin-10 in Health and Disease. Fillatreau S. O’Garra A., eds. Springer Berlin Heidelberg, Berlin, Heidelberg, p. 157–190. 10.1007/978-3-662-43492-5_8 25004818

[34] Xu J. Yang Y. Qiu G. Lal G. Wu Z. Levy D. E. Ochando J. C. Bromberg J. S. Ding Y. 2009 c-Maf regulates IL-10 expression during Th17 polarization. J. Immunol. 182: 6226–6236. 19414776 10.4049/jimmunol.0900123 PMC2834209

[35] Pot C. Jin H. Awasthi A. Liu S. M. Lai C.-Y. Madan R. Sharpe A. H. Karp C. L. Miaw S.-C. Ho I. C. Kuchroo V. K. 2009 Cutting edge: IL-27 induces the transcription factor c-Maf, cytokine IL-21, and the costimulatory receptor ICOS that coordinately act together to promote differentiation of IL-10-producing Tr1 cells. J. Immunol. 183: 797–801. 19570826 10.4049/jimmunol.0901233 PMC2768608

[36] Kubo M. Motomura Y. 2012 Transcriptional regulation of the anti-inflammatory cytokine IL-10 in acquired immune cells. Front. Immunol. 3: 275. 22969768 10.3389/fimmu.2012.00275 PMC3430973

[37] Tudor C. Marchese F. P. Hitti E. Aubareda A. Rawlinson L. Gaestel M. Blackshear P. J. Clark A. R. Saklatvala J. Dean J. L. E. 2009 The p38 MAPK pathway inhibits tristetraprolin-directed decay of interleukin-10 and pro-inflammatory mediator mRNAs in murine macrophages. FEBS Lett. 583: 1933–1938. 19416727 10.1016/j.febslet.2009.04.039 PMC4798241

[38] Yu H. Sun Y. Haycraft C. Palanisamy V. Kirkwood K. L. 2011 MKP-1 regulates cytokine mRNA stability through selectively modulation subcellular translocation of AUF1. Cytokine 56: 245–255. 21733716 10.1016/j.cyto.2011.06.006 PMC3185122

[39] Németh Z. H. Lutz C. S. Csóka B. Deitch E. A. Leibovich S. J. Gause W. C. Tone M. Pacher P. Vizi E. S. Haskó G. 2005 Adenosine augments IL-10 production by macrophages through an A2B receptor-mediated posttranscriptional mechanism. J. Immunol. 175: 8260–8270. 16339566 10.4049/jimmunol.175.12.8260 PMC2000336

[40] Povoleri G. A. M. Lalnunhlimi S. Steel K. J. A. Agrawal S. O’Byrne A. M. Ridley M. Kordasti S. Frederiksen K. S. Roberts C. A. Taams L. S. 2020 Anti-TNF treatment negatively regulates human CD4^+^ T-cell activation and maturation in vitro, but does not confer an anergic or suppressive phenotype. Eur. J. Immunol. 50: 445–458. 31722123 10.1002/eji.201948190 PMC7079027

[41] Gaba A. Grivennikov S. I. Do M. V. Stumpo D. J. Blackshear P. J. Karin M. 2012 Cutting edge: IL-10-mediated tristetraprolin induction is part of a feedback loop that controls macrophage STAT3 activation and cytokine production. J. Immunol. 189: 2089–2093. 22865915 10.4049/jimmunol.1201126 PMC3424405

[42] Aschenbrenner D. Foglierini M. Jarrossay D. Hu D. Weiner H. L. Kuchroo V. K. Lanzavecchia A. Notarbartolo S. Sallusto F. 2018 An immunoregulatory and tissue-residency program modulated by c-MAF in human T_H_17 cells. [Published erratum appears in 2019 *Nat. Immunol.* 20: 109.] Nat. Immunol. 19: 1126–1136. 30201991 10.1038/s41590-018-0200-5 PMC6402560

[43] Urbano P. C. M. Aguirre-Gamboa R. Ashikov A. van Heeswijk B. Krippner-Heidenreich A. Tijssen H. Li Y. Azevedo V. F. Smits L. J. T. Hoentjen F. 2018 TNF-α-induced protein 3 (TNFAIP3)/A20 acts as a master switch in TNF-α blockade-driven IL-17A expression. J. Allergy Clin. Immunol. 142: 517–529. 29248493 10.1016/j.jaci.2017.11.024

[44] Kim H. S. Jang S. W. Lee W. Kim K. Sohn H. Hwang S. S. Lee G. R. 2017 PTEN drives Th17 cell differentiation by preventing IL-2 production. J. Exp. Med. 214: 3381–3398. 29018045 10.1084/jem.20170523 PMC5679178

[45] Luo J. Ming B. Zhang C. Deng X. Li P. Wei Z. Xia Y. Jiang K. Ye H. Ma W. 2018 IL-2 inhibition of Th17 generation rather than induction of treg cells is impaired in primary Sjögren’s syndrome patients. Front. Immunol. 9: 1755. 30150979 10.3389/fimmu.2018.01755 PMC6100298

[46] Kasahara T. Hooks J. J. Dougherty S. F. Oppenheim J. J. 1983 Interleukin 2-mediated immune interferon (IFN-gamma) production by human T cells and T cell subsets. J. Immunol. 130: 1784–1789. 6403613

[47] Reem G. H. Yeh N. H. 1984 Interleukin 2 regulates expression of its receptor and synthesis of gamma interferon by human T lymphocytes. Science 225: 429–430. 6429853 10.1126/science.6429853

[48] Lin J.-X. Li P. Liu D. Jin H. T. He J. Ata Ur Rasheed M. Rochman Y. Wang L. Cui K. Liu C. 2012 Critical Role of STAT5 transcription factor tetramerization for cytokine responses and normal immune function. Immunity 36: 586–599. 22520852 10.1016/j.immuni.2012.02.017 PMC3551341

[49] Naundorf S. Schröder M. Höflich C. Suman N. Volk H.-D. Grütz G. 2009 IL-10 interferes directly with TCR-induced IFN-γ but not IL-17 production in memory T cells. Eur. J. Immunol. 39: 1066–1077. 19266486 10.1002/eji.200838773

[50] Sievers Q. L. Petzold G. Bunker R. D. Renneville A. Słabicki M. Liddicoat B. J. Abdulrahman W. Mikkelsen T. Ebert B. L. Thomä N. H. 2018 Defining the human C2H2 zinc finger degrome targeted by thalidomide analogs through CRBN. Science 362: eaat0572. 30385546 10.1126/science.aat0572 PMC6326779

[51] Hung K. H. Su S. T. Chen C. Y. Hsu P. H. Huang S. Y. Wu W. J. Chen M. J. M. Chen H. Y. Wu P. C. Lin F. R. 2016 Aiolos collaborates with Blimp-1 to regulate the survival of multiple myeloma cells. Cell Death Differ. 23: 1175–1184. 26823144 10.1038/cdd.2015.167 PMC4946885

[52] Awwad M. H. S. Kriegsmann K. Plaumann J. Benn M. Hillengass J. Raab M. S. Bertsch U. Munder M. Weisel K. Salwender H. J. 2018 The prognostic and predictive value of IKZF1 and IKZF3 expression in T-cells in patients with multiple myeloma. OncoImmunology 7: e1486356. 30288348 10.1080/2162402X.2018.1486356 PMC6169592

