# Supplementary material for: IKZF3/Aiolos Is Associated with but Not Sufficient for the Expression of IL-10 by CD4+ T Cells
Source: J Immunol. 2020 Apr 22;204(11):2940–8. doi: 10.4049/jimmunol.1901283 (PMC7231851; doi:10.4049/jimmunol.1901283)
Supplement: Data Supplement [file JI_1901283.zip › JI_1901283_Supplemental_Figures_1.pdf]

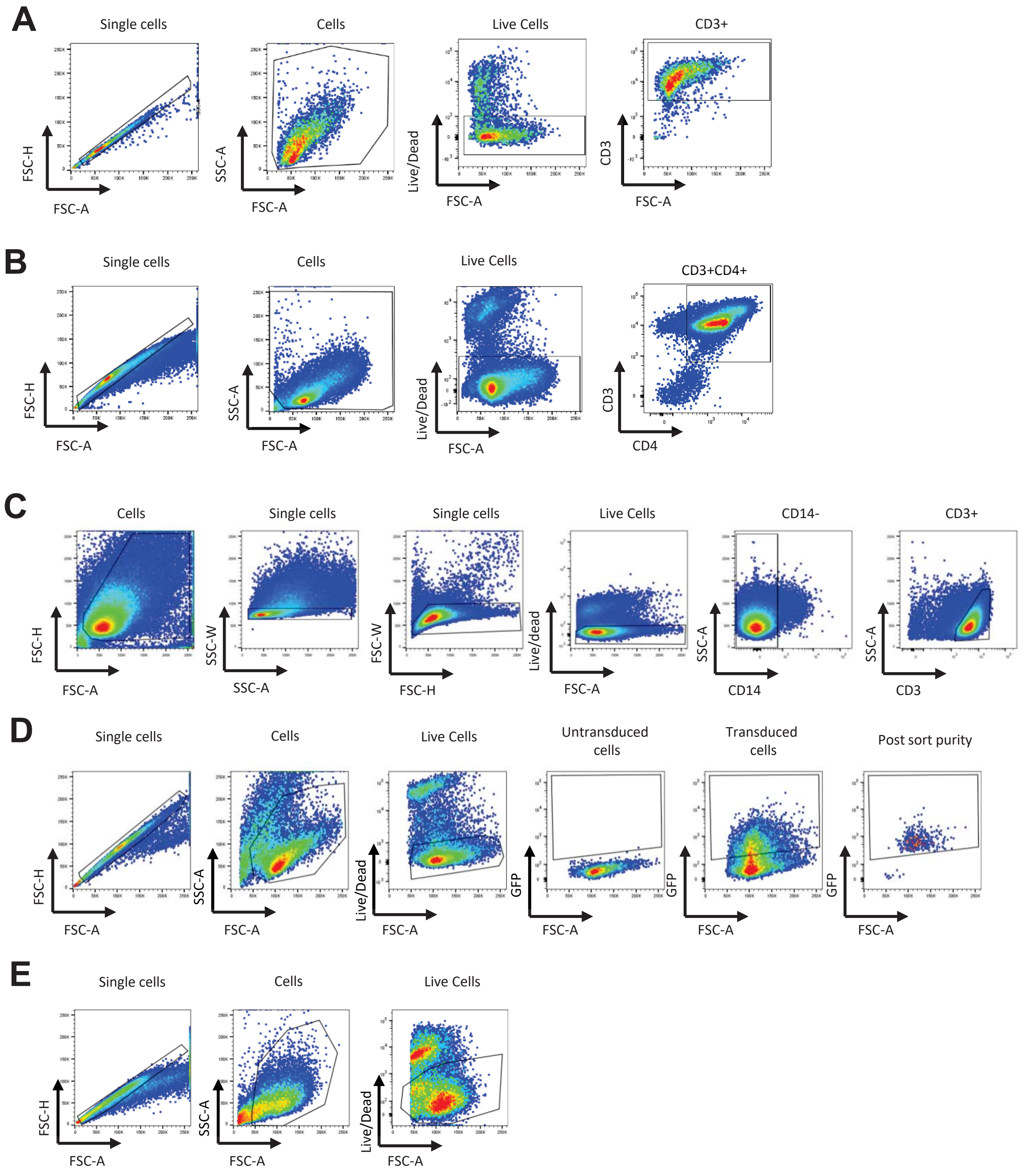

Supplemental Figure 1. Gating strategy for flow cytometry data. (A, B) CD4+ T cells were isolated from the peripheral blood of healthy donors and gated on live (A) CD3+ events or (B) CD3+ CD4+. This gating strategy was used in Figures 1A and B, 2A-F, 3B-D, Supplemental Figure 2B, Supplemental Figure 3A and B. (C) CD4+ T cells were isolated from the peripheral blood of healthy donors and cultured at a 1:1 ratio with autologous CD14+ monocytes and gated on Live CD14- CD3+ Events. This gating strategy was used in Supplemental Figure 2A. (D) CD4+ T cells were stimulated and transduced with lentiviral particles for 7 days and then sorted on Live GFP+ CD3+ events by a FACS ARIA. The depicted gating strategy was used in the analysis of Figure 4C and D. (E) HEK293T cells were transfected with plasmids encoding IKZF3 or cMAF as well as GFP. These cells were subsequently stained for viability, IKZF3 and cMAF and gated on Live cell events. This gating strategy was used in Supplemental Figure 4A and B.

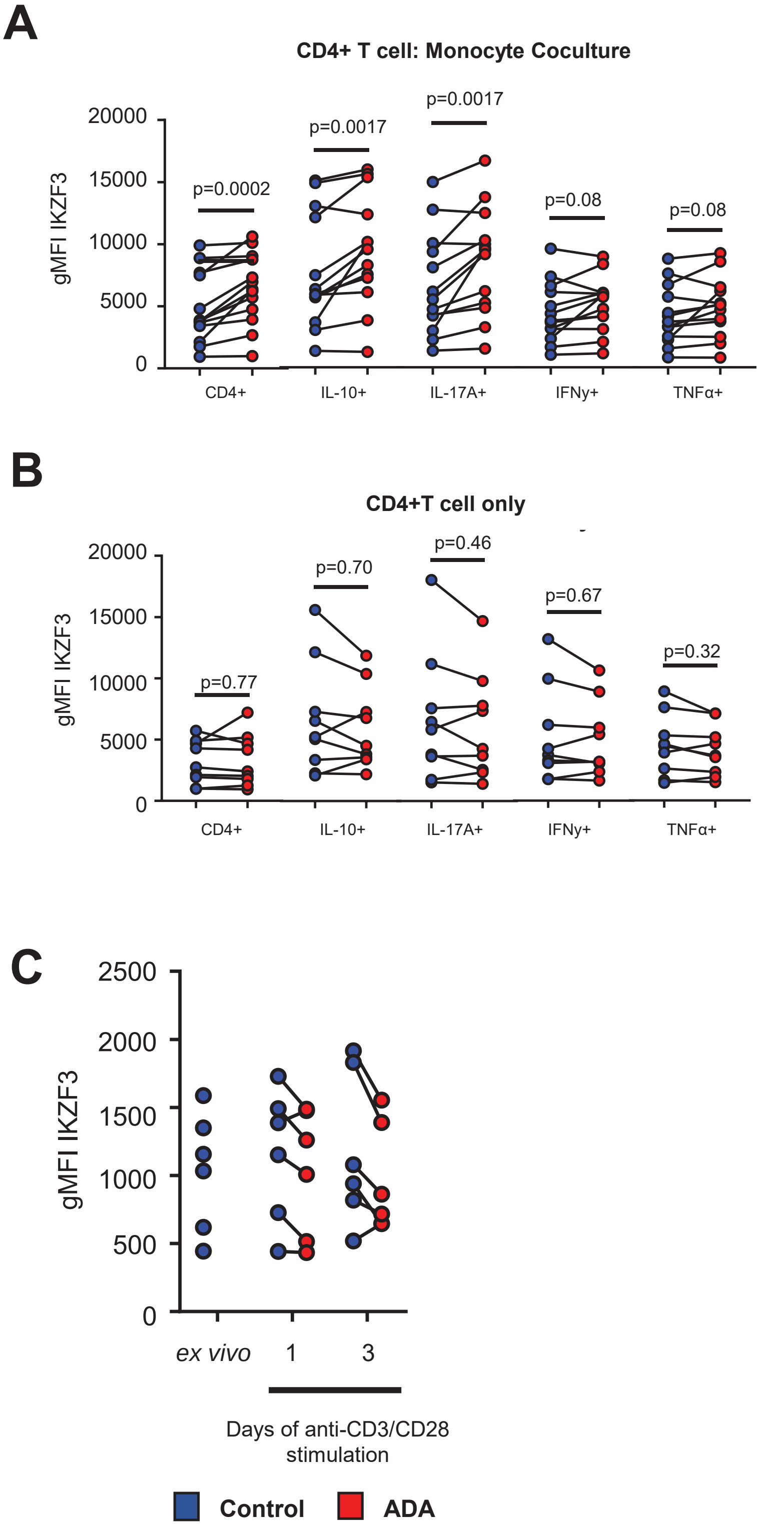

Supplemental Figure 2. TNF blockade does not alter IKZF3 expression in CD4+ T cells in the absence of monocytes. (A) CD4+ T cells and CD14+ monocytes from healthy donors were cultured together at a 1:1 ratio for 3 days with anti-CD3 mAb in the absence (blue circles) or presence (red circles) of 1 µg/mL adalimumab. Cells were restimulated with PMA and ionomycin and assessed for cytokine and IKZF3 expression (n=13). (B) Primary CD4+ T cells were stimulated with anti-CD3/CD28 mAb for 3 days in the absence or presence of 1µg/mL adalimumab. Cells were restimulated with PMA and ionomycin and assessed for cytokine and IKZF3 expression (n= 9). (C) Primary CD4+ T cells were stimulated with anti-CD3/CD28 mAb for 0, 1 or 3 days in the absence or presence of 1µg/mL adalimumab and assessed for IKZF3 expression (n=6). Data in A and B analysed by Wilcoxon test, data in C analysed by Friedman test.

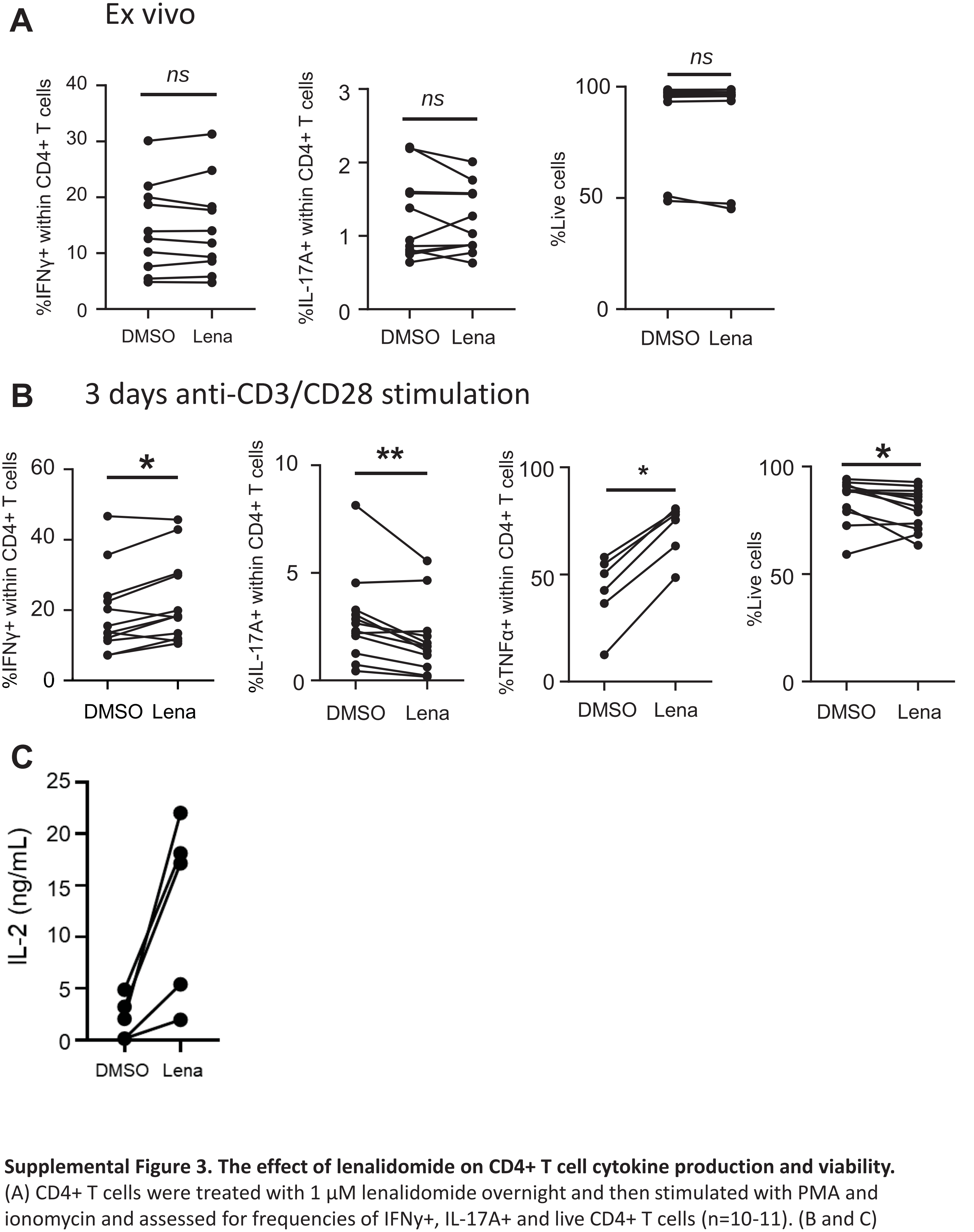

Supplemental Figure 3. The effect of lenalidomide on CD4+ T cell cytokine production and viability. (A) CD4+ T cells were treated with 1 μM lenalidomide overnight and then stimulated with PMA and ionomycin and assessed for frequencies of IFNγ+, IL-17A+ and live CD4+ T cells (n=10-11). (B and C) CD4+ T cells were stimulated with anti-CD3/CD28 mAb for 3 days in the presence of 1 μM lenalidomide or DMSO control and (B) subsequently restimulated with PMA and ionomycin and assessed for IFNγ+, IL-17A+, TNF+ and live CD4+ T cells (by staining with Live/Dead discriminator dye). (C) Cell culture supernatants of CD4+ T cells treated with DMSO or lenalidomide were assessed for IL-2 secretion (n=5). Data analysed by Wilcoxon test.

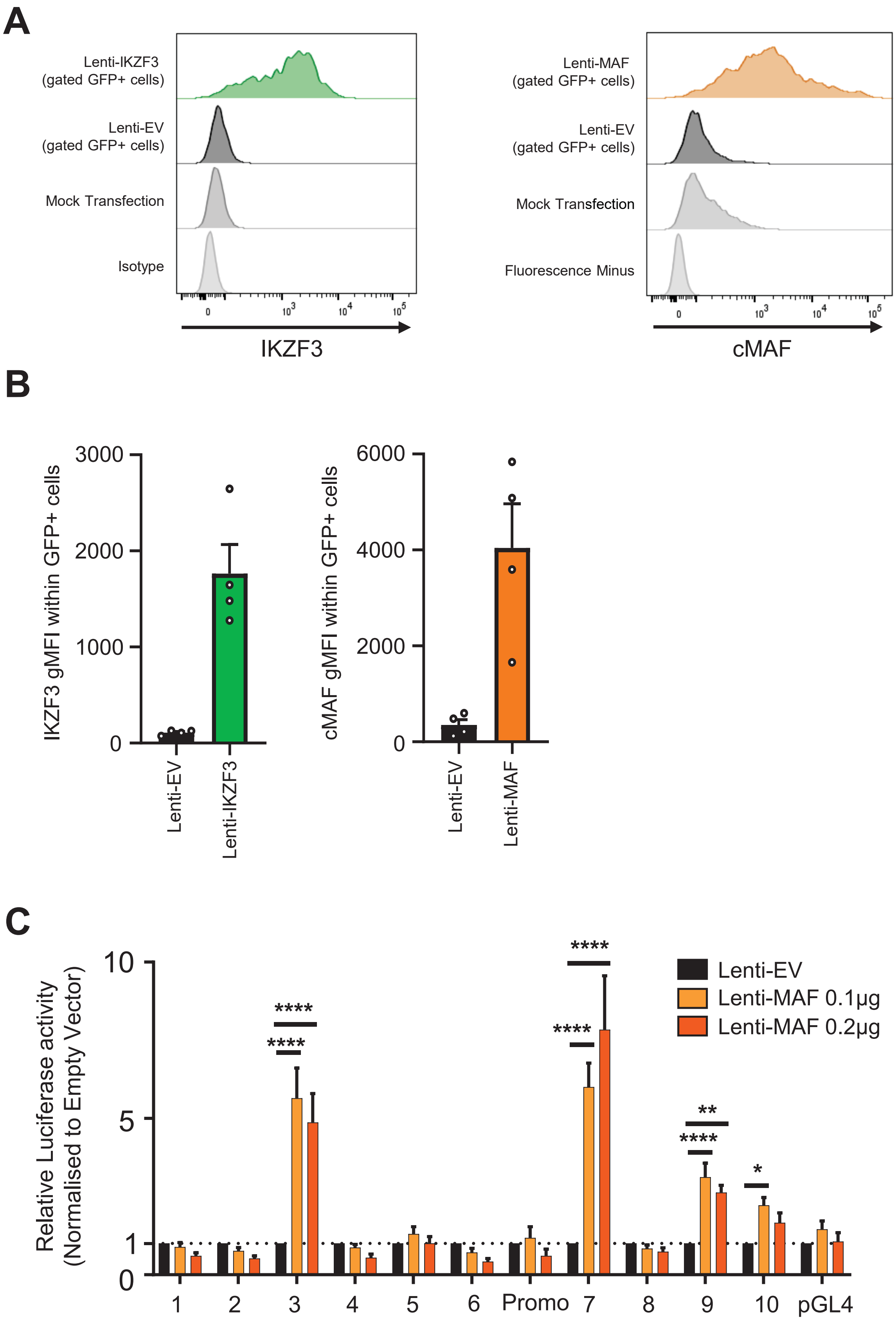

Supplemental Figure 4. Overexpression of proteins in HEK293T cells. (A,B) HEK293T cells were transfected with 2 μg of Lenti-IKZF3, Lenti-EV or Lenti-MAF for 48 hours and assessed for IKZF3 or cMAF expression by flow cytometry. (A) Representative histograms and (B) cumulative data (n=4) are shown. (C) HEK293T cells were transfected with the putative IL10 enhancer and promoter reporter plasmids in addition to Lenti-EV or Lenti-MAF (n=4). Data in C analysed by 2-way ANOVA with multiple comparisons.
